# Effect of Chromatin Structure on the Extent and Distribution of DNA Double Strand Breaks Produced by Ionizing Radiation; Comparative Study of hESC and Differentiated Cells Lines

**DOI:** 10.3390/ijms17010058

**Published:** 2016-01-02

**Authors:** Priyanka Venkatesh, Irina V. Panyutin, Evgenia Remeeva, Ronald D. Neumann, Igor G. Panyutin

**Affiliations:** Department of Radiology and Imaging Sciences, Clinical Center, National Institutes of Health, Bethesda, MD 20892, USA; priy.venkatesh@gmail.com (P.V.); ipanyutinv@mail.cc.nih.gov (I.V.P.); remeeva@gmail.com (E.R.); rneumann@mail.cc.nih.gov (R.D.N.)

**Keywords:** ionizing radiation, human embryonic stem cells, chromatin structure

## Abstract

Chromatin structure affects the extent of DNA damage and repair. Thus, it has been shown that heterochromatin is more protective against DNA double strand breaks (DSB) formation by ionizing radiation (IR); and that DNA DSB repair may proceed differently in hetero- and euchromatin regions. Human embryonic stem cells (hESC) have a more open chromatin structure than differentiated cells. Here, we study the effect of chromatin structure in hESC on initial DSB formation and subsequent DSB repair. DSB were scored by comet assay; and DSB repair was assessed by repair foci formation via 53BP1 antibody staining. We found that in hESC, heterochromatin is confined to distinct regions, while in differentiated cells it is distributed more evenly within the nuclei. The same dose of ionizing radiation produced considerably more DSB in hESC than in differentiated derivatives, normal human fibroblasts; and one cancer cell line. At the same time, the number of DNA repair foci were not statistically different among these cells. We showed that in hESC, DNA repair foci localized almost exclusively outside the heterochromatin regions. We also noticed that exposure to ionizing radiation resulted in an increase in heterochromatin marker H3K9me3 in cancer HT1080 cells, and to a lesser extent in IMR90 normal fibroblasts, but not in hESCs. These results demonstrate the importance of chromatin conformation for DNA protection and DNA damage repair; and indicate the difference of these processes in hESC.

## 1. Introduction

Increased chromatin density has been shown to have a protective effect against radiation damage in cell survival assays and in experiments regarding induction of DNA damage [1,2,3,4]. Chromatin has been characterized as existing in roughly two states: highly compacted heterochromatin, and less compacted, transcriptionally active euchromatin [5]. One possible mode of protection is that chromatin proteins may act as radical scavengers, reacting with unstable radical species produced by radiation [6]. These proteins also push water molecules away from tightly packaged DNA, perhaps further mitigating damage from ionized hydroxyl radicals. Other studies have demonstrated how chromatin density is linked with radioresistance and increased survival [4]. For example, histone deacetylase (HDAC) inhibitors, which de-condense chromatin structure, have been shown to radiosensitize tumors and various cancer cell lines [7,8,9]. These studies demonstrated that cells with more heterochromatin might be more resilient against ionizing radiation damages than those with more euchromatin. These observations may have interesting implications for the mechanisms leading to DNA damage in embryonic and adult stem cells, which have been shown to have comparatively more open chromatin structures than their differentiated counterparts [5,10].

It is generally agreed that quickly regenerating tissues such as bone marrow, skin and intestine are the most sensitive to radiation in regards to damage and potential carcinogenesis [11]. Given the long life span and continuous proliferation of the adult stem cells responsible for long-term homeostasis of these tissues, these cells may be especially prone to accumulating and multiplying harmful mutations. Embryonic stem cells hold enormous potential for cell therapy, but have still not been well characterized with regard to potential hazards. Both types of cells may be exposed to environmental radiation and radiation from routine medical imaging tests such as positron emission tomography (PET), and computed tomography (CT) studies. It is important to determine the risk to stem cells for developing potentially harmful mutations before they are used in therapy for patients, as well as to better understand the risk of normal tissue and adult stem cells when exposing patients to radiation.

Embryonic stem (ES) cells are defined as having the ability to differentiate into cells of all three germ layers, and to proliferate indefinitely. The genome of ES cells is in a highly malleable state, so that these cells may eventually enter into any direction of differentiation. This is manifested as very open, unwound euchromatin with relatively little transcriptionally silent heterochromatin [5]. Even stem cells present in normal adult tissues, such as hematopoetic stem cells, have a comparatively more open and plastic chromatin structure relative to their differentiated progeny [12]. After differentiation, there is an observed condensation of chromosome territories, and an increase in heterochromatin foci due to the silencing of unnecessary genes [10]. Given the aforementioned role of heterochromatin in providing stability to DNA, the relatively high levels of euchromatin in ES cells may render them more vulnerable to damage from ionizing radiation.

The impact of heterochromatin on DNA damage, repair, and radiosensitivity in terms of cell survival has been studied extensively [1,2]. However the relationship between chromatin structure and the actual induction of DNA damage has been given less attention. Studies suggested that heterochromatin slows down the efficiency of DNA repair, because chromatin may need to relax and unwind so that repair proteins are able to access the DNA damage site [4]. Other studies showed that cell treatment with histone deacetylase (HDAC) inhibitors resulted in radiosensitization of these cells [8,9].

In this study, we wished to determine whether human embryonic stem cells (hESC) are inherently more susceptible to the formation of double strand breaks compared to several types of control cells. These controls included cells differentiated from the hESC lines, terminally differentiated IMR90 fibroblasts, and HT080 cancer cells. We also sought to determine whether these differences were a result of differences in heterochromatin density, and whether there was a difference in the formation of repair foci between these cell types. We show that there appear to be considerably higher numbers of DSBs in pluripotent cells in response to high doses of ionizing radiation. At the same time, the number of repair centers formed was comparable between cell lines. We also found that in hESC DNA repair foci localized almost exclusively outside the heterochromatin regions. Unexpectedly, there appeared to be global, dose-dependent changes in certain epigenetic modifications related to heterochromatin (H3K9me3 and H3K27me3) in HT1080 and IMR90 cells, but not in hESC.

## 2. Results

### 2.1. hESC Have Less Heterochromatin than Partially and Fully Differentiated Cells

Previous studies have shown hESC contain more euchromatin than fully differentiated cells (reviewed in [5]). Pluripotent cells maintain a dynamic, more open and euchromatin rich state, which increasingly shifts towards heterochromatin as cells differentiate and silence various genes [13,14]. To verify this in our cell cultures, we stained H9 and H14 hESC, HT1080 fibrosarcoma cells, and IMR90 primary fibroblasts for heterochromatin marker H3K9me3. We also differentiated our H9 and H14 hESC towards the endodermal lineage using activin A and bFGF as previously described [15], to directly compare these cells to the differentiated lineages.

**Figure 1:**
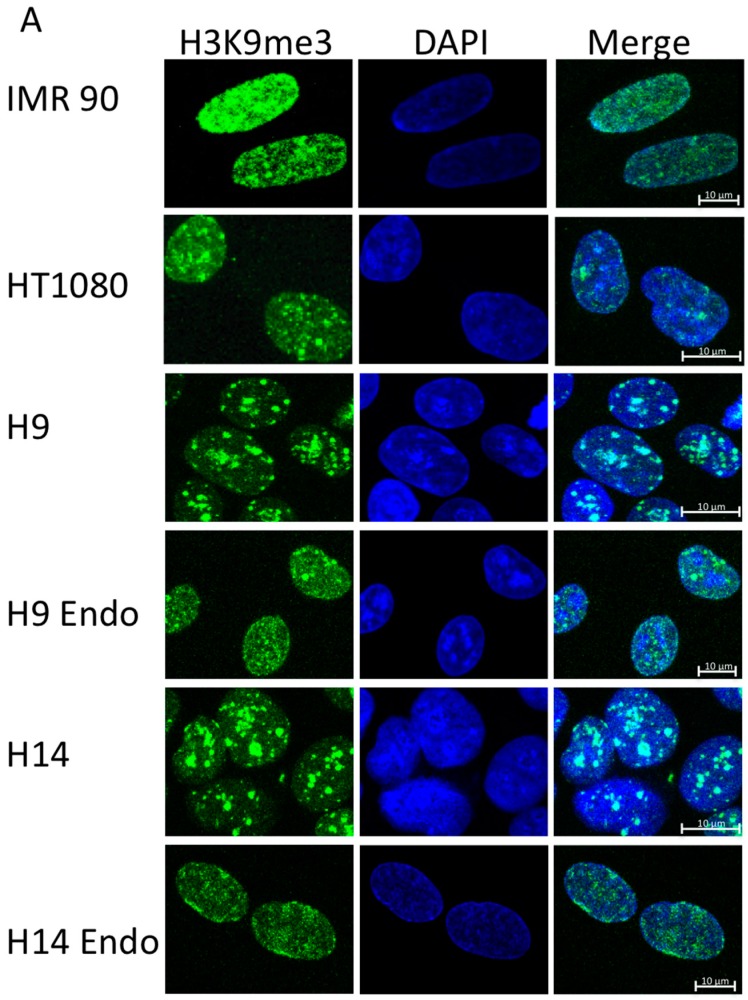

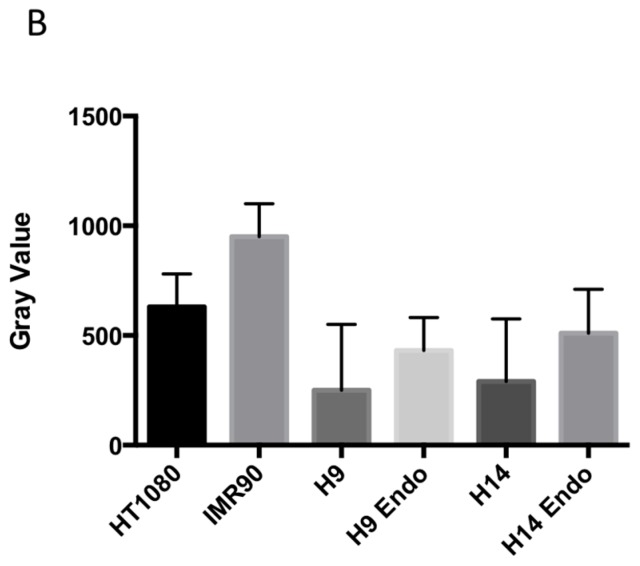
(**A**) Immunofluorescent microscopy staining of various cell lines for H3K9me3 (**green**) and DAPI (**blue**), intersecting regions show as **cyan** on the merged images; (**B**) Fluorescence intensity analysis of H3K9me3 staining in various cell lines. Scale bars are 10 µm.

Staining with H3K9me3 antibody shows that the structure of heterochromatin in hESC is different than that of differentiated cells or cancer cells (Figure 1A). The staining pattern in hESC shows distinct, heterogeneous in size, bright speckles that coincide with brighter regions of 4′,6-diamidino-2-phenylindole (DAPI) staining, giving us an additional indication that this marker is localizing to compact heterochromatin. In contrast, differentiated cells, IMR90, and HT1080 cells have more evenly distributed heterochromatin throughout the nucleus. However, HT1080 cells show some granular heterochromatin foci in addition to higher levels of overall staining compared to H9 and H14 hESC. IMR90 fibroblasts show the highest levels of overall H3K9me3 staining (Figure 1B).

Staining of H9 and H14 cells differentiated into the endodermal lineage with H3K9me3 antibody is almost uniform and resembles that of IMR90 cells (Figure 1). The bright speckles of heterochromatin staining observed in parental pluripotent hESC disappear with endodermal differentiation. These results confirm that differentiation of hESC results in rearrangement in chromatin structure.

**Figure 2:**
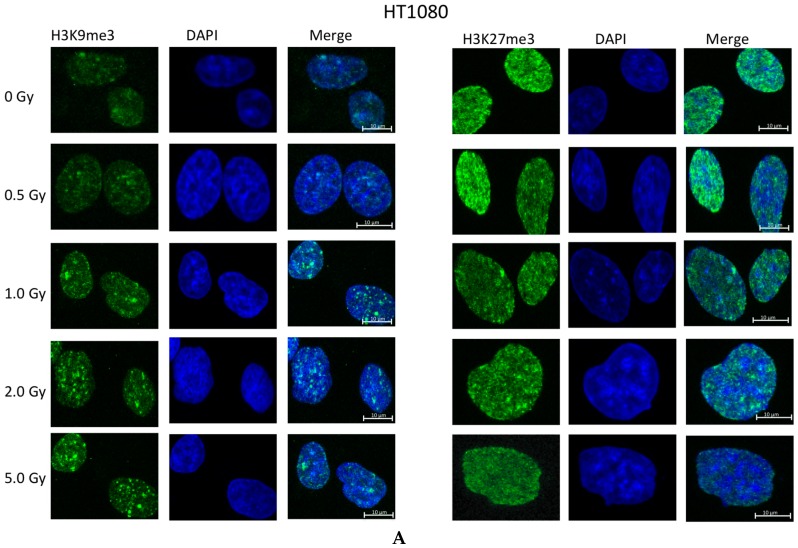

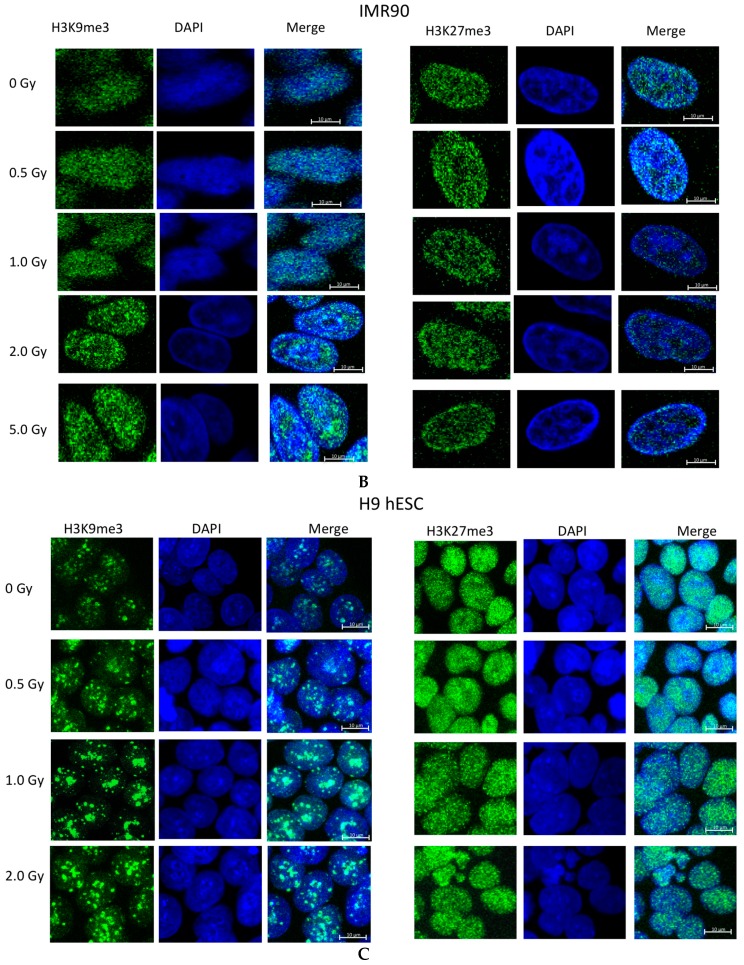

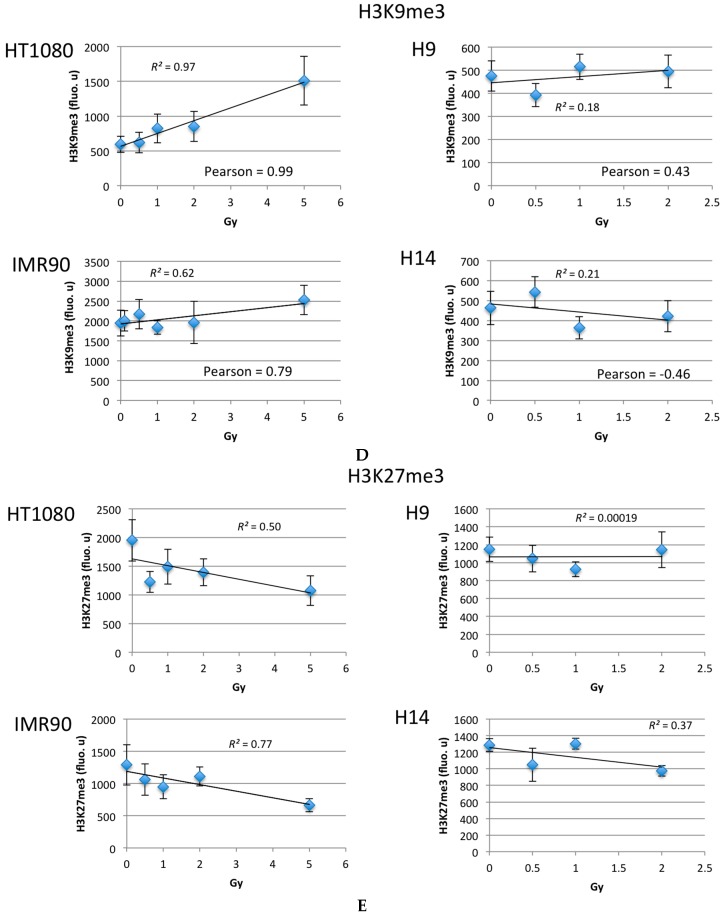
Immunofluorescent microscopy staining for H3K9me3 (**green**, left panels) and H3K27me3 (**green**, right panels), DAPI (**blue**) of (**A**) HT1080; (**B**) IMR90; (**C**) H9 hES cell lines exposed to various doses of IR; 0 Gy is the sham-exposed control. Intersecting regions of green and blue staining show as **cyan** on the merged images; scale bars are 10 µm. Dependence of staining intensity (arbitrary fluorescent units) for (**D**). H3K9me3 and (**E**) H3K27me3 from exposure dose (Gy) for various cell lines (indicated). The slopes of the best-fit linear regression line and Pearson’s correlation coefficients are shown on the graphs. HT1080 H3K9me3 expression: slope is significantly non zero (*p* < 0.01); HT1080: H3K27me3 expression: slope is non zero (not significant, *p* = 0.13); IMR90 h3k9me3: *p* = 0.25; IMR90 h3k27me3: *p* = 0.05.

We also stained cells with another heterochomatin marker H3K27me3. This staining is more evenly distributed throughout the nucleus for all cell types (Figure 2A–C, top panels). The brighter regions of H3K27me3 staining does not correspond to the regions with more intense DAPI staining, suggesting that they do not co-localize with condensed silent heterochromatin, but possibly to the more flexible form of facultative heterochromatin [5].

### 2.2. Ionizing Radiation Dose Dependent Change in Heterochromatin Staining

We then studied the effect of ionizing radiation on distribution of heterochromatin. IMR90 and HT1080 cells, and H9 and H14 hESC cells were exposed to different doses of radiation. The highest exposure dose was slightly lower for hESC (2 Gy) than for IMR90 and HT1080 cells (5 Gy) because of the higher radiosensitivity of the former. All cell lines at all dose points were stained for H3K9me3 and H3K27me3 markers 20 min after irradiation to allow chromatin modifications to take place (Figure 2A–C, images for H14 cells are not shown). Images were quantitated as described in the Experimental Section. HT1080 cells show an increase in H3K9me3 staining intensity after exposure to radiation in a dose-dependent manner (Figure 2D). The slope of increase in fluorescent signal as a function of dose for HT1080 was significantly different from zero (*p* < 0.05). The H3K9me3 staining for IMR90 appeared also to be increasing, but the slope was not quite statistically significant (*p* = 0.07). Fluorescent intensity measurements of H3K9me3 staining after exposure to ionizing radiation showed no significant change for in H9 and H14 hESC lines (Figure 2D). Staining for H3K27me3 decreased with increase of the dose of IR for HT1080 cells (*p* = 0.13), and significantly decreased for IMR90 cells (*p* = 0.05) (Figure 2E). For H14 hESC the decrease in H3K27me3 staining was less pronounced, while H9 hESC showed no change in H3K27me3 staining with increase of IR dose (Figure 2E).

### 2.3. Time Dependent Recovery of HT1080 Cells after Exposure to Ionizing Radiation

**Figure 3:**
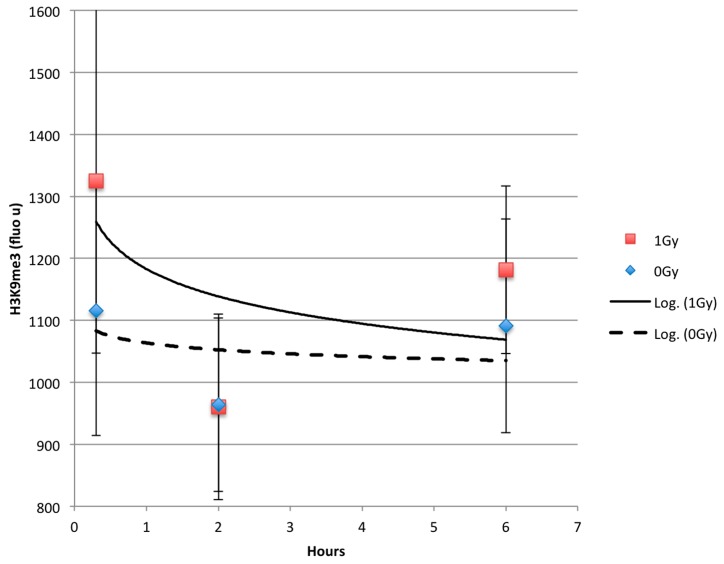
Dependence of staining intensity (arbitrary fluorescent units) from time after exposure to 1 Gy of IR for and sham-exposed (0 Gy) HT1080 cells. Logarithmic regression lines for 1 Gy (**solid**) and 0 Gy (**dashed**) data points are shown.

To determine whether the change in H3K9me3 expression was transient or more permanent, HT1080 cells were exposed to 0 or 1 Gy of radiation and fixed after 20 min, 2 h, and 6 h of recovery. Cells were stained for H3K9me3. Quantification of fluorescence showed an initial increase in fluorescence for H3K9me3 after 20 min of cells exposed to 1 Gy IR compared to control cells (Figure 3). This effect practically disappeared by 2 h of recovery after exposure. Although we had seen that higher radiation doses resulted in a more significant increase in H3K9me3 staining (Figure 2D); radiation doses above 1 Gy resulted in a significant cell death, making measurements of H3K9me3 staining signal unreliable [16].

### 2.4. hESC Show More Double Strand Breaks after Exposure to High Doses of Ionizing Radiation

**Figure 4:**
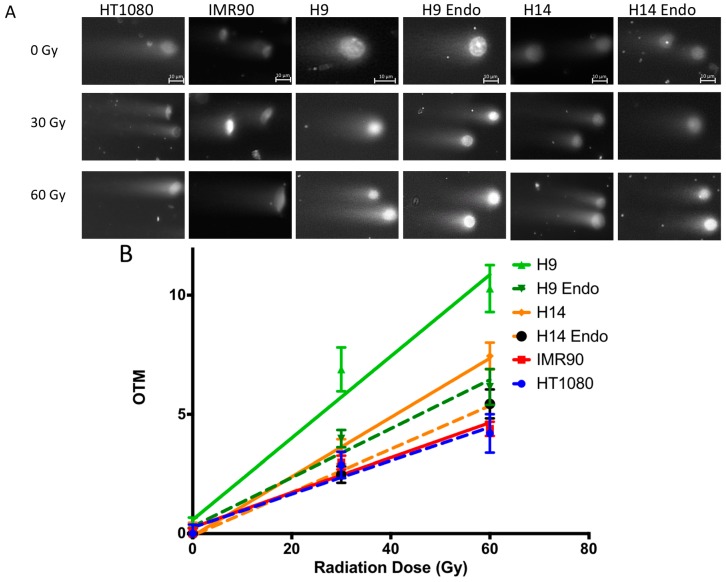
Neutral comet assay. (**A**) Examples of comets after 60 Gy IR; (**B**) Dependence of comet’s olive tail moments (OTM) from IR dose after background subtraction (normalized to 0 OTM at 0 Gy). *N* > 50 comets for each dose point and cell type were analyzed as described in the Methods. The background OTM at 0 Gy was subtracted from 30 and 60 Gy measurements. Error bars represent standard errors of the mean (SEM). Data points were fitted with linear regression lines; *R*^2^ value for all curves was significant (>95%). OTM of H9 hESC was significantly higher than that of H9 endoderm differentiated cells (*p* < 0.01), HT1080 fibrosarcoma cells (*p* < 0.01), and IMR90 primary fibroblasts (*p* < 0.01). The OTM of H14 hESC was higher than that of H14 endoderm differentiated cells (*p* = 0.23), and significantly higher than the OTM of HT1080 cells (*p* < 0.01) and IMR90 cells (*p* < 0.01).

To determine whether stem cells are more susceptible to DNA double strand breaks from ionizing radiation than differentiated cells, we performed the neutral comet assay as previously described [17,18]. H9 and H14 hESC, endoderm differentiated H9 and H14, HT1080, and IMR90 cells were exposed to 0, 30, or 60 Gy of gamma radiation. Exposure to these higher doses than that in the previous experiments was required because of considerably lower sensitivity of the comet assay. Slides were scored for the Olive Tail Moment (OTM, the product of the tail length and percent DNA in the tail), which is proportional to the number of double strand breaks [18] (Figure 4A). H9 hES cells had significantly higher OTMs than differentiated H9 cells (*p* < 0.01), terminally differentiated fibroblast cell line IMR90 (*p* < 0.01), and HT1080 fibrosarcoma cells (*p* < 0.01) as shown in Figure 4B. H14 hESC had higher OTMs than differentiated H14 (*p* = 0.23), and significantly higher OTMs than IMR90 (*p* < 0.01) and HT1080 cells (*p* < 0.01) (Figure 4B). Thus, DNA of hESC acquire more double strand breaks than DNA of differentiates cell lines exposed to the same dose of IR.

### 2.5. hESC Show Comparable Numbers of Radiation Induced Foci between Cell Lines after Exposure to Low Doses of IR

**Figure 5:**
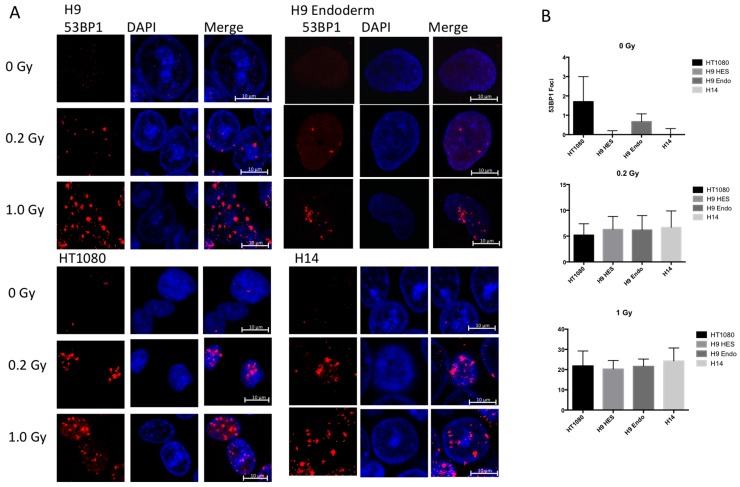
53BP1 foci formation after IR exposures. (**A**) Maximum intensity projection confocal microscopy images staining with 53BP1 antibody (**red**) and DAPI (**blue**); (**B**) Measured numbers of foci per cells for various cell lines after exposure to 0, 0.2 and 1.0 Gy.

We also tested whether there were more 53BP1 radiation induced foci (RIF) in pluripotent stem cells compared to differentiated cells and HT1080 cells after exposure to 0, 0.2, and 1.0 Gy of ionizing radiation. While this is more reflective of the DNA damage response mechanism in the different cells lines than the actual number of induced double strand breaks, we were curious as to whether more RIF were able to form in the euchromatin-rich hESC. The results are shown in Figure 5. Without irradiation, HT1080 and H9 differentiated cells show some background RIF, while H9 and H14 hESC were almost clear of them. In all cell types the number of 53BP1 RIF increase linearly with radiation dose at almost equivalent rates, despite the fact that significant differences in the amount of DSB were observed between the two embryonic clones and the more differentiated IMR90 and HT080 cells. Therefore, even though there were more DNA double strand breaks in hESC than in differentiated and cancer cells lines they show similar number of RIF per cell at the same dose of IR.

**Figure 6:**
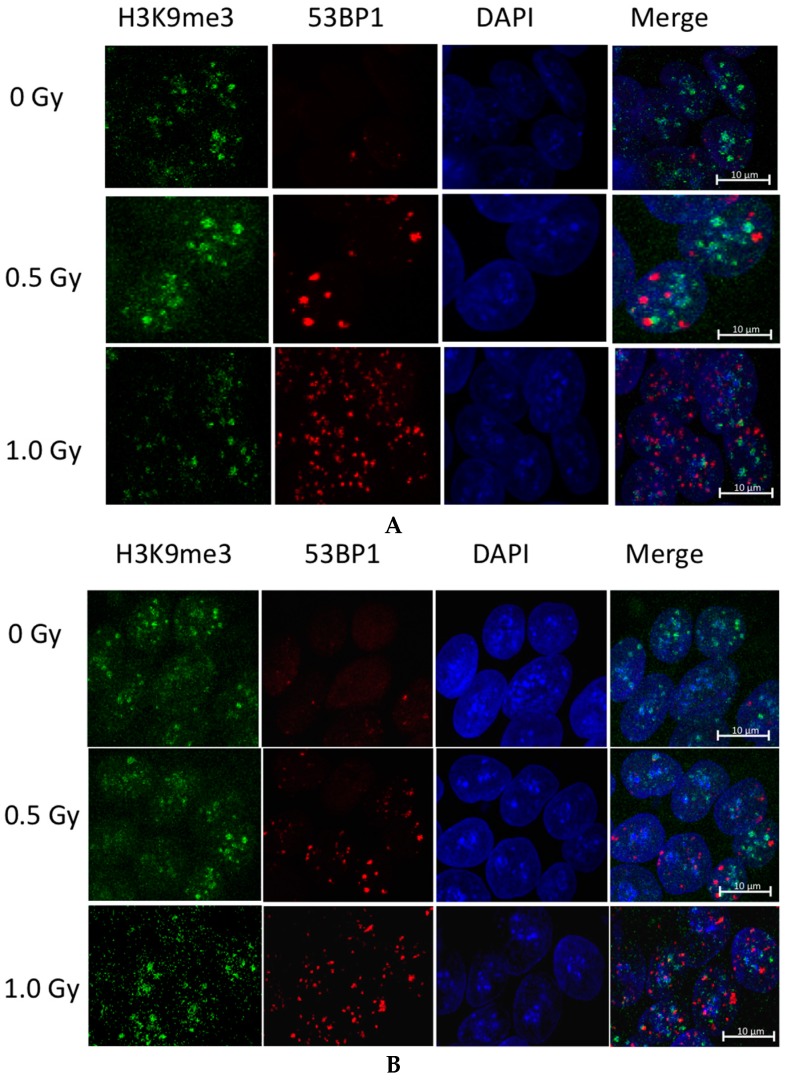
Co-staining for heterochromatin and RIF. Maximum intensity projection confocal microscopy images of (**A**) H9 and (**B**) H1 hESC staining for H3K9me3 (**green**); 53BP1 (**red**) and DAPI (**blue**). Majority of the RIF located outside heterochromatin regions.

We also co-stained H9 and H1 hESC after exposure to IR with H3K9me3 heretochromatin marker, and 53BP1 marker of DNA repair foci. Results presented in Figure 6 show that DNA repair foci almost never coincide with the hererochromatin regions in hESC. This was confirmed using “colocalization” function of Zeiss Zen software that measures Pearson correlation coefficients of signals in different channels. The correlation coefficients of green (H3K9me3 staining) and red (53BP1 staining) channels were *r*_1_ = 0.11 and *r*_2_ = 0.13 for H9 and H1 hESC correspondingly. That proves the lack of co-localization of RIF and heterochromatin regions.

## 3. Discussion

One of the functions of chromatin is to protect and stabilize DNA. Mechanisms of this stabilization can include protection from hydroxyl radicals [3,19,20] as well as reduced access of soluble clastogens to DNA. Other studies have linked increased chromatin density to decreased radiosensitivity as evidenced by cell survival assays after treatment with HDAC inhibitors, and increased heterochromatin in response to 3D cell growth [4,7,8,9]. There is also evidence that chromatin organization affects regional mutation rates in human cancer cells [21].

Herein we tested whether stem cells, which have comparatively less compacted chromatin than differentiated cells, are more vulnerable to DSB induction. We were also interested in seeing if there was a global chromatin response to IR. To do this, we compared induction of double strand breaks, 53BP1 repair foci, and changes in heterochromatin staining after radiation exposure of cell lines. We compared H9 hESC, H14 hESC, IMR90 primary fibroblasts, and HT1080 fibrosarcoma cells. Additionally, we directly compared H9 and H14 hESC with their differentiated progeny to assess the difference in damage and repair mechanisms before and after differentiation.

### 3.1. hESC Have Less Heterochromatin than Lineage-Committed Cells

Previous studies have shown an increase in heterochromatin after differentiation, and higher amounts of heterochromatin in terminally differentiated cell lines compared with pluripotent cell lines [5,13]. One study comparing the chromatin-modification profiles between hESCs and IMR90 fibroblasts found that nearly one third of the genome differs in regard to its chromatin structure, and a significant number of the changes arose due to H3K9me3 and H3K27me3 marks [14]. In particular, they found that chromatin regions marked by repressive modifications, such as H3K9me3 and H3K27me3, are considerably larger in lineage-restricted human lung fibroblasts when compared to hESC.

To confirm these previous results, we chose to examine the heterochromatin markers H3K9me3, which is tightly associated with silent heterochromatin [22], and H3K27me3, which is generally associated with facultative heterochromatin [23]. Our results confirmed that H9 and H14 hESC had the least amount of H3K9me3 compared to other cell types. Differentiated hESC and HT1080 cells had higher extent of heterochromatin staining, and IMR90 fibroblasts had the greatest amount of staining. 

### 3.2. Global Changes in Chromatin Methylation after Exposure to IR

Previous studies have shown that exposure to ionizing radiation results in different responses of the DNA-chromatin complex. It was shown that there is a localized de-compaction at DSB sites in heterochromatin, to allow access of repair proteins to the site of damage [24,25]. At the same time, there is evidence of global compaction of chromatin in response to DNA damage [26]. A study examining DNA damage responsive histones found a decrease in H3K9Ac and H3K56Ac, both of which are associated with euchromatin; thereby suggesting a decrease in euchromatin in response to damage [27]. We found that H3K9me3 staining increases in HT1080 cells and IMR90 cells after exposure to IR (Figure 2). H3K27me3 staining appeared to decrease in both HT1080 and IMR90 cells after exposure to IR (Figure 2).

While the H3K9me3 enrichment might be explained by chromatin condensation in order to protect DNA, the reasons for H3K27me3 to diminish are unclear. As H3K27me3 is fairly evenly distributed, it was difficult to ascertain whether there was a localized reduction of this marker around 53BP1 repair foci. However, the distinct regions of H3K9me3 staining in hESC did make it possible for a co-localization analysis. 53BP1 foci clearly did not overlap with regions of H3K9me3 staining (Figure 6).

As the pluripotent state of hESC are strictly maintained, any reorganization of their chromatin structure could lead to a reduction in plasticity [13], and may compromise their ability to properly differentiate. If stem cells indeed modify their chromatin in response to radiation, this would likely induce differentiation [28]. Yet, a number of studies have shown that the pluripotency of stem cells is not significantly altered after exposure to ionizing radiation [29,30,31]. Another possibility is that H3K9me3 plays direct role in DNA DSB repair. Thus, it was recently found that the activity of Tip60 acetyltransferase could only be activated at DSB in regions of high H3K9me3 density, such as heterochromatin [32]. Alternatively, H3K9 methylation may be transiently increased at DSBs in regions of low H3K9me3 density to allow for Tip60 activation. Clearly, the question of molecular mechanisms behind RIF and H3K9me3 anti-colocalization requires further investigation.

## 4. Experimental Section

### 4.1. Cell Culture

HT1080 Human fibrosarcoma cells and IMR 90 fibroblasts were obtained from ATCC (Manassas, VA, USA) and were cultured according to ATCC recommendations. H1, H9 (WiCell, Madison, WI, USA), and H14 Human Embryonic Stem Cells (provided by Barbara Mallon, NIH Stem Cell Unit, Bethesda, MD, USA) were grown in feeder-free conditions using BD Matrigel hESC-qualified Matrix (San Jose, CA, USA). The culture was maintained as previously described [30].

### 4.2. Directed Endoderm Differentiation

Human ESCs were seeded onto 6-well plates covered with BD Matrigel human ESC-qualified Matrix (BD Biosciences, East Rutherford, NJ, USA) at approximately 10^5^ cells per well. Then, the cells were maintained in mTeSR1 medium at 5% CO_2_ and 37 °C for two days with medium changed each day. Starting from day 3, cells in culture were maintained in differentiation media: DMEM/F12 (Stemcell Technologies, Vancouver, BC, Canada) supplemented with 5% KSR (knockout serum replacement) (Invitrogen, Grand Island, NY, USA), 100 ng/mL Activin A (Stemcell Technologies), 4 ng/mL bFGF as was described previously in [15].

### 4.3. Irradiation

For comet assay samples, cells were collected using Trypsin–EDTA treatment, and irradiated in suspension in PBS with a Gamma-Cell 220 (Atomic Energy of Canada, Ltd., Ottawa, ON, Canada, now known as Best Theratronics) at a dose rate of 9.8 Gy/min. Cells were irradiated on ice at 30 and 60 Gy, and were kept on ice post-IR.

For immunostaining, cells were grown in BD-Falcon culture slides. HT1080 and IMR 90 cells were grown to 50% confluency. Human ESC cells were grown on culture slides coated with BD Matrigel for 2 days before IR. Slides were irradiated using Eldorado 8 ^60^Co teletherapy unit (MDS Nordion, Ottawa, ON, Canada, formerly Atomic Energy of Canada, Ltd.; dose rate about 1 Gy/min). Cells were allowed to recover for 20 min before fixation.

### 4.4. Neutral Comet Assay

The neutral comet assay allows for sensitive detection of DNA double strand breaks [17]. Fragmented DNA as a result of DSBs migrates out of the cells when placed under electrophoresis. The amount of DNA in the resulting “tail” is proportional to the number of double strand breaks. Cells were irradiated and brought into suspension as described above. The assay was prepared as described in [18]. Electrophoresis was run on the slides at 1 V/cm for 30 min. Slides were stained for 20 min in a staining solution of 2.5 µg/mL propidium iodide in distilled water. Slides were imaged using a Zeiss Axiovert 200 microscope, and images were analyzed using Image J software (NIH, Bethesda, MD, USA). The following parameters were measured: total intensity, tail length, and percent DNA in tail; then the tail moment of *N* = 50 cells were scored per slide as described in [18]. For comparison between cell lines the background OTM at 0 Gy was subtracted from 2 other measurements. Reported values are the mean ± standard error of the mean (SEM).

### 4.5. Immunostaining and Imaging

Slides were fixed in ice-cold methanol for 10 min, and subsequently stored in ethanol at −20 °C for up to one week before staining. Slides were subsequently immunostained according to the protocol described in [30]. Cells were stained with primary antibodies anti-53BP1 (rabbit), H3K9me3 (mouse) or H3K27me3 (mouse) (Millipore, Billerica, MA, USA). Cells were counterstained with secondary antibodies anti-mouse Alexa-fluor 488 and anti-rabbit Alexa-fluor 555 (Invitrogen). Cell nuclei were stained with DAPI. Images were acquired at a constant exposure and laser intensity using an upright laser scanning confocal microscope (series 710, Carl Zeiss, Thornwood, NY, USA) using Plan-Apochromat objectives (20 air, Numerical Aperture (NA) 1⁄4 0.8; 63 oil-immersion, NA 1⁄4 1.4). Excitation for DAPI (blue channel), AlexaFluor488 (green channel), and AlexaFluor546 (red channel) was performed using laser lines at 405, 488, and 561 nm, respectively, and images were acquired sequentially to minimize crosstalk. Measurements were made using Zeiss Zen software. The fluorescent intensities of individual cells in green and red channels were measured using masks obtained in the blue channel, *i.e.*, only inside nuclei. The reported values are the average intensity of all cells (*N* > 20) in a dose point ± standard deviation (SD). Scale bars in some images were added retroactively to illustrate differences in magnifications of different panels.

53BP1 foci were counted manually. *N* > 20 cells were counted for each dose point. To avoid subjective counts of very small foci, a constant threshold was applied to confocal stacks so that binary images of medium and large sized foci could be counted in 3D. Co-localization of 53BP1 and H3K9me3 antibody staining was performed using “colocalization” function of Zeiss Zen software.

## 5. Conclusions

We found that heterochromatin in hESC is confined to distinct regions; while in differentiated cells it is distributed more evenly within the nuclei. Using the comet assay we showed that the same dose of ionizing radiation produced considerably more DSB in hESC than in their differentiated derivatives, normal human fibroblasts, and a cancer cell line. At the same time, the number of DNA repair foci was not statistically different among these cells. We also found that in hESC, DNA repair foci localized almost exclusively outside the heterochromatin regions. We demonstrated that exposure to ionizing radiation resulted in an increase in heterochromatin marker H3K9me3 in cancer HT1080 cells, and to a lesser extent in normal fibroblasts, but not in hESCs. These results demonstrate the importance of chromatin conformation for DNA protection, and DNA damage repair; and indicate the difference of these processes in hESC.
